# Aboriginal Population and Climate Change in Australia: Implications for Health and Adaptation Planning

**DOI:** 10.3390/ijerph19127502

**Published:** 2022-06-19

**Authors:** Jeffrey C. Standen, Jessica Spencer, Grace W. Lee, Joe Van Buskirk, Veronica Matthews, Ivan Hanigan, Sinead Boylan, Edward Jegasothy, Matilde Breth-Petersen, Geoffrey G. Morgan

**Affiliations:** 1 Health Protection NSW, St Leonards, NSW 2065, Australia; jessica.spencer@health.nsw.gov.au (J.S.); g.lee@sydney.edu.au (G.W.L.); 2 School of Public Health, Faculty of Medicine and Health, University of Sydney, Camperdown, NSW 2006, Australia; joseph.vanbuskirk@sydney.edu.au (J.V.B.); ivan.hanigan@sydney.edu.au (I.H.); sinead.boylan@csiro.au (S.B.); edward.jegasothy@sydney.edu.au (E.J.); matilde.petersen@sydney.edu.au (M.B.-P.); geoffrey.morgan@sydney.edu.au (G.G.M.); 3 University Centre for Rural Health, Faculty of Medicine and Health, University of Sydney, Lismore, NSW 2480, Australia; veronica.matthews@sydney.edu.au

**Keywords:** Aboriginal health, Aboriginal population, adaptation, climate exposure, climate and health, climate vulnerability, equity, health policy, indigenous health

## Abstract

The health impacts of climate are widely recognised, and extensive modelling is available on predicted changes to climate globally. The impact of these changes may affect populations differently depending on a range of factors, including geography, socioeconomics and culture. This study reviewed current evidence on the health risks of climate change for Australian Aboriginal populations and linked Aboriginal demographic data to historical and projected climate data to describe the distribution of climate-related exposures in Aboriginal compared to non-Aboriginal populations in New South Wales (NSW), Australia. The study showed Aboriginal populations were disproportionately exposed to a range of climate extremes in heat, rainfall and drought, and this disproportionate exposure was predicted to increase with climate change over the coming decades. Aboriginal people currently experience higher rates of climate-sensitive health conditions and socioeconomic disadvantages, which will impact their capacity to adapt to climate change. Climate change may also adversely affect cultural practices. These factors will likely impact the health and well-being of Aboriginal people in NSW and inhibit measures to close the gap in health between Aboriginal and non-Aboriginal populations. Climate change, health and equity need to be key considerations in all policies at all levels of government. Effective Aboriginal community engagement is urgently needed to develop and implement climate adaptation responses to improve health and social service preparedness and secure environmental health infrastructure such as drinking water supplies and suitably managed social housing. Further Aboriginal-led research is required to identify the cultural impacts of climate change on health, including adaptive responses based on Aboriginal knowledges.

## 1. Introduction

Climate change is a complex global phenomenon that influences environmental conditions that, in turn, affect human health [1,2]. Although it is difficult to attribute accurately the magnitude of health impacts caused by the anthropogenic change in the climate, evidence of health changes can nonetheless be inferred from the more robust data that links particular climate-related hazards with health outcomes [1]. The pathways by which climate change can affect health have been well described: extreme events such as bushfires, floods and storms can inflict injury and death directly, whilst heatwaves can increase cardiovascular morbidity and mortality [2,3]. Climate change can degrade environmental and ecological systems and affect human health indirectly: warmer conditions can encourage pathogen proliferation that causes water and food-borne diseases; increased sources of air pollutants such as bushfire smoke and dust can exacerbate respiratory diseases; ecological environments can become more conducive to mosquito-borne disease transmissions; and droughts can become more severe leading to food and water insecurity [2,3,4,5]. The social, economic and demographic disruptions caused by climate change can adversely impact livelihoods, including mental health and social and emotional well-being [2,3].

In Australia, an observed 1.4 °C of warming since 1910 has already caused more severe heatwaves and fire weather, reduced rainfall and severe droughts in parts of Australia [6]. People with existing climate-sensitive conditions, who are poorly resourced and living in areas more severely impacted by climate extremes, are most affected by climate change [5]. In Australia, this includes Aboriginal and Torres Strait Islander communities. The term “Aboriginal” is used to describe the original inhabitants of Australia and their descendants. The term “Torres Strait Islander” is used to describe the original inhabitants and their descendants from the Torres Strait Islands located to the north of mainland Australia. As the focus of this study is on NSW, “Aboriginal” is respectfully used in an inclusive way to refer to all Aboriginal and Torres Strait Islander people residing in NSW.

Aboriginal people have historically been managing land and water resources sustainably for ongoing farming practices, drinking water and cultural practices [7]. As the natural environment is increasingly impacted by climate change, Aboriginal communities will continually be disproportionately affected due to their close physical and spiritual relationships with their Country (traditional homelands) and dependence on land and water resources. Climate change is seen as another component of ongoing colonisation, where Aboriginal people have been dispossessed, communities decimated and culture suppressed [8,9]. Aboriginal people bear greater proportions of ill health, including cardiovascular and respiratory diseases, diabetes and mental health conditions [10,11] and poorer status across the social determinants, including education, employment, income and housing conditions, compared with non-Aboriginal populations [12].

Climate change also presents an opportunity for redress. Aboriginal communities are humanity’s oldest continuing culture, having adapted to gradual and abrupt changes over millennia, including colonisation [13]. The application of Aboriginal knowledges and cultural practices has shown enormous environmental, health and well-being benefits. This has been demonstrated by Aboriginal rangers participating in caring for Country activities showing lower rates of obesity, diabetes and cardiovascular diseases [14,15]. Resourcing and empowering Aboriginal communities to lead place-based climate adaptation and mitigation processes will be critical to addressing current and future climate challenges in Australia.

New South Wales (NSW) is the fifth largest and most populated state on the continent, situated on the east coast of Australia with an area of 801,137 km^2^ and a coastline of 2101 km [16]. NSW is geographically diverse, influencing climate and population patterns. Temperatures get progressively hotter moving inland towards the west and north-west of the state, where the north-west receives highly variable rainfall and very high temperatures [17]. The elevated mountainous areas of the Great Dividing Range separate the east coast from the inland. The range enhances rainfall near the coast, where temperatures are moderate [17]. The flat, western side of the range covers two-thirds of the state and includes much of the state’s agricultural land, with inland rivers forming part of the Murray–Darling River network flowing towards South Australia. In 2021, 8,186,800 residents lived in major cities, regional centres and rural areas across NSW [18]. In 2016, one-third of Australia’s Aboriginal people were estimated to live in NSW (*n* = 265,685, 33%) and within NSW, 46.3% were estimated to live in major cities (*n* = 123,099) [19]. However, as a proportion, Aboriginal people in NSW are more likely to live outside of major urban areas than non-Aboriginal populations and some in discrete Aboriginal communities—former reserves and missions that are now designated Aboriginal land title and mostly located in rural and regional locations.

Temperatures in NSW are predicted to warm by 0.7 °C by 2030 and 2.1 °C by 2070 based on a medium CO_2_ emissions scenario [17,20]. The number of days greater than 35 °C is expected to increase by approximately 20 and 40 extra days per year in coastal and north-western NSW, respectively, by 2070 [17]. Fire weather is expected to increase in western NSW in spring and summer, whilst rainfall is projected to decrease in spring and increase in autumn with regional variations [17].

This study reviewed current evidence on the health risks of climate change for Aboriginal populations in NSW and described historical and future exposure to a range of climate-related factors in the Aboriginal compared to non-Aboriginal populations. Guided by Aboriginal partner organisations, this study further identified key challenges and issues associated with adaptation to these climate exposures.

## 2. Materials and Methods

Project partners from Aboriginal government and non-government agencies working in the NSW Aboriginal community and health sectors guided the development and implementation of the study. Detailed population data on usual resident populations (URPs), based on counts from the Australian Bureau of Statistics (ABS) 2016 Census for Population and Housing, were obtained [21]. The ABS divides Australia into Statistical Areas of different levels based on population size. URPs were obtained for Statistical Areas Level 1 (SA1), which contain between 200 and 800 usual residents [22]. To account for spatial variability of exposures within SA1s, ABS 2016 population grids [23] were extracted by SA1 and were used to calculate population weighted estimates by SA1. URPs are known to undercount Aboriginal populations in Australia, and so estimates are aggregated to the state level with overall proportions reported. While the ABS estimated resident populations (ERPs) have been corrected for the known undercounting of Aboriginal people [24], these population data are only available at the Statistical Area 2 (SA2) level, a spatial unit many times larger than the smaller SA1 spatial unit (containing between 3000 and 25,000 people). In order to assess the suitability of using URPs versus ERPs, estimates were obtained using both methods in a sensitivity analysis, with negligible differences in proportions found across methods. As such, the smaller geographical unit (i.e., SA1 level geography using URP estimates) was used for the primary analyses.

Discrete Aboriginal communities across NSW were also mapped using data identified by various government programs and compiled by the NSW Health Environmental Health Branch. The data have been validated by on-site field visits and mapping using the G-NAF (Geocoded National Address File), a trusted index of Australian address information. G-NAF contains the state, suburb, street, number and coordinate reference (or “geocode”) for street addresses in Australia. G-NAF does not contain any personal information or details relating to an individual or business [25].

### 2.1. Historical and Projected Climate Data

Selected historical climate exposure data were obtained from the NSW Department of Planning, Industry and Environment (DPIE) through the Australian Bureau of Meteorology (BoM) Australian Water Availability Project (AWAP) [26] for NSW at a resolution of 25 km by 25 km over the 1990 to 2019 period. All data were aggregated to annual averages for the region. Future predictions for data on selected climate parameters were sourced from the NSW and ACT Climate Modelling (NARCliM) project. The NARCliM modelling was able to draw from global climate model outputs and downscaled them to finer 10 km by 10 km resolutions for the period from 2020 to 2039 [20,27]. This study selected key climate-related exposures based on publicly available data relevant to the NSW context, including heat, rainfall, drought and fire danger.

NSW climate data were obtained from the AWAP [26] to estimate population exposures to extreme heat and heatwaves. Average annual days with daily mean temperatures of 35 °C or above were calculated, as well as an estimate of annual average maximum heatwave duration in days, as estimated by the Excess Heat Factor metric [28]. This metric identifies the longest heatwave duration where the day’s rolling three-day mean temperature both exceeds the 95th percentile of historical temperatures and the recent 30-day average for that region [28].

Rainfall variability and average rainfall estimates were obtained for NSW from AWAP [26]. Daily rainfall estimates were used to calculate annual averages. Rainfall variability was also calculated as the 90th rainfall percentile minus the 10th rainfall percentile divided by the 50th rainfall percentile (i.e., median). This metric provides an indication of how much rainfall varies from low to extreme.

Drought exposure was calculated using the Standard Precipitation Evapotranspiration Index with a six-month rolling sum of rainfall, and evapotranspiration, between 1950 and 2020, sourced from the AWAP. In this method, cumulative six-month estimates of precipitation were fitted to a standard gamma probability distribution with z scores calculated for each six-monthly rolling sum. Negative z scores indicate drought, with values of −1.3 or lower indicating at least moderate drought. Evapotranspiration was estimated using the Thornthwaite method due to a lack of windspeed data [29]. The proportion of months in drought per period was compared between the period of 1990 to 2006 and 2007 to 2020 to provide an estimate of the relative increase in drought-affected months between these two periods.

Bushfire vulnerability was estimated using the McArthur Forest Fire Danger Index (FFDI), a composite measure of the area’s drought factor, daily maximum temperature, daily afternoon humidity and wind speed [30]. Higher values of FFDI indicate a higher likelihood of fires starting and spreading, with values between 25 and 49 indicating very high danger and values of 50 or over indicating severe fire danger. Historical (1990–2009) and projected FFDI values were sourced from NARCliM.

### 2.2. Statistical Analysis

Exposure grids for each of the climate metrics were averaged across ABS Statistical Area 1 (SA1) boundaries for 2016 [22]. Population-weighted exposure estimates were then calculated by SA1, with categories informed by the distribution of each exposure. Aboriginal and non-Aboriginal populations were then summed by exposure category for each population. In order to calculate the odds of each population living in areas with a higher risk exposure, exposure estimates were further categorised into “high” versus “lower” risk, with dichotomisation informed by the categories identified by the distributions. For example, average annual days over 35 °C were categorised into <5 days; 5–<10 days; 10–<20 days; 20–<50 days and 50+ days above 35 °C. This categorisation was dichotomised to <20 days indicating “lower risk” and 20 or greater days above 35 °C indicating “high risk”. Odds ratios were calculated by comparing the proportion of Aboriginal populations living in areas classified as high risk with non-Aboriginal populations living in those same areas, using the Wald method with confidence intervals calculated accordingly.

As a sensitivity analysis, odds ratios were stratified by the ABS Index of Relative Socioeconomic Disadvantage (IRSD) quintile [31] to assess whether differential exposures could be explained by socioeconomic status. The IRSD is a general socioeconomic index that includes measures of the relative disadvantage of people and households within an area, such as low-income or low-skilled occupations. A low score indicates a relatively greater disadvantage [32].

All data processing and visualisation were performed using RStudio (Version 4.1.3) (RStudio Inc., Boston, MA, USA, 2019). This study used publicly available data on climate modelling and Aboriginal demographics.

## 3. Results

**Figure 1:**
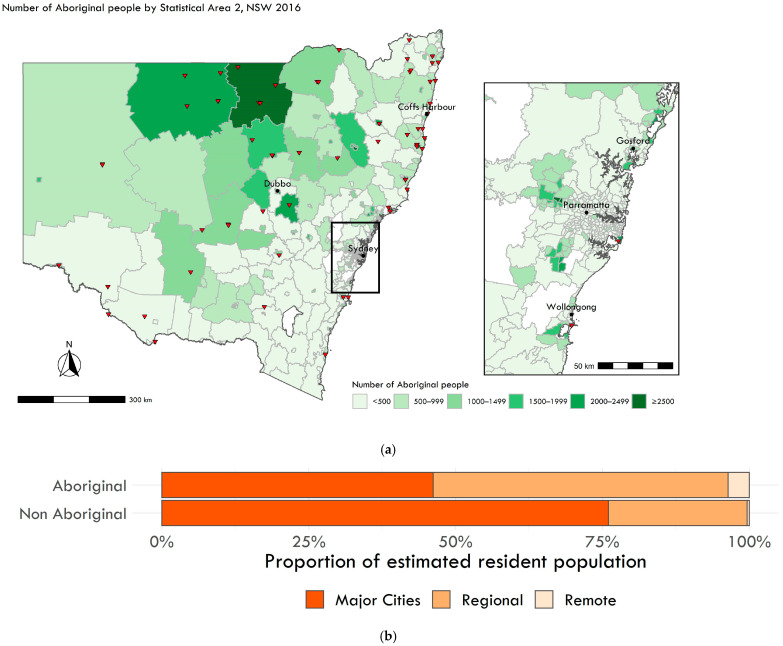
Distribution of Aboriginal people in NSW, 2016: (**a**) Number of Aboriginal people by Statistical Area 2 in NSW and the Greater Sydney Regions, 2016. Triangle markers indicate discrete Aboriginal communities with populations between 10–1000 people. (**b**) Proportion of Aboriginal versus non-Aboriginal populations by rurality. Source: Australian Bureau of Statistics [22] and NSW Ministry of Health, Environmental Health Branch.

Figure 1 shows the distribution of Aboriginal populations and locations of discrete Aboriginal communities in NSW. Most Aboriginal people (46%) lived in major cities; however, Aboriginal people made up a greater proportion of the population in regional and remote areas.

### 3.1. Comparison of Climate Exposure Levels between Aboriginal and Non-Aboriginal Populations

Across all climate indicators, higher proportions of Aboriginal populations compared to non-Aboriginal populations were found to live in areas with more extreme climate-related exposures. All climate exposure risk indicators are summarised by exposure categories in Table 1, with additional descriptive statistics included in Appendix A Table A1.

For periods of excessive heat (i.e., the average of the longest annual heatwave duration), 26% of NSW Aboriginal populations lived in areas with seven or greater maximum number of heatwave days annually, compared with 9% of non-Aboriginal populations (OR 3.79, 95% CI 3.75–3.83). Aboriginal populations had 4.3 times the odds of living in areas with a high number of annual average days exceeding 35 °C (20% vs. 5%; 95% CI 4.3 to 4.4) and were predicted to have 3.5 times the odds of living in areas with five or more additional days exceeding 35 °C in the next 20 years (32% vs. 12%; 95% CI 3.4 to 3.5).

For rainfall variability, 13% of the Aboriginal population in NSW lived in areas with moderate, moderate to high or high rainfall variability (11%, 1% and 1%, respectively), compared with 3% of the non-Aboriginal population in NSW (3%, 0.1% and 0.2%, respectively). That is, Aboriginal populations had 4.7 times the odds of living in areas with higher exposure to rainfall variability (95% CI 4.6 to 4.7) and likely more prone to drought or flooding issues. For rainfall, 36% of the Aboriginal population in NSW lived in areas with less than 800 mm annual average rainfall (4% ≤400 mm and 32% between 400 and less than 800 mm) compared with 17% of non-Aboriginal populations (2% ≤400 mm and 15% between 400 and less than 800 mm; OR 2.77, 95% CI 2.75 to 2.80). Aboriginal populations were also more likely to live in areas with projected decreases in rainfall, with 23% of Aboriginal populations living in these areas, compared with 12% of non-Aboriginal populations in the next 20 years (OR 2.1, 95% CI 2.1 to 2.2).

Aboriginal populations were 4.3 times (95% CI 4.2 to 4.3) more likely to live in areas with an average of three or more days annually where Forest Fire Danger Index (FFDI) exceeded 50 (extreme fire danger) compared with non-Aboriginal populations (20% vs. 6%). In addition, Aboriginal populations were predicted to have 4.4 times (95% CI 4.3 to 4.4) the odds of living in areas with 0.5 or greater additional days annually where FFDI exceeds 50 in the next 20 years (12% vs. 3%).

Aboriginal people were also more likely to live in areas with increased months of drought between 2007 and 2020 compared with the previous period, 1990–2006. Specifically, Aboriginal people were 2.4 times (95% CI 2.3 to 2.4) more likely to live in areas with increases in drought-affected months than non-Aboriginal people (32% vs. 17%). Drought frequency and intensity were expected to increase in the future; however, future drought changes were not included in this analysis due to the level of uncertainty in its projection.

**Figure 2:**
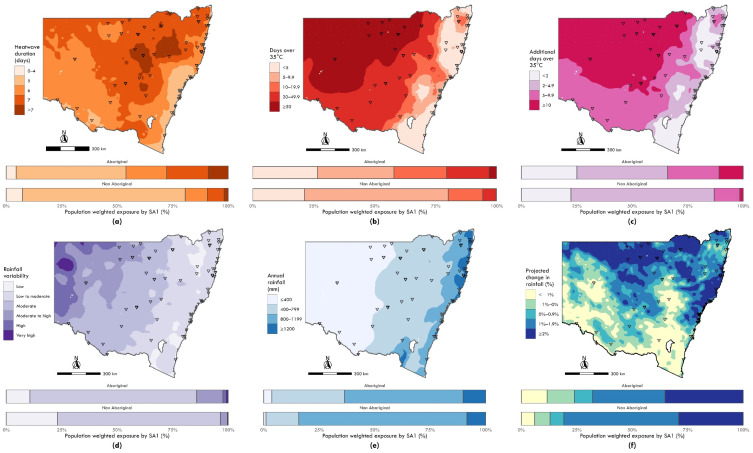
Maps of climate exposures with bar charts indicating relative exposure by category across Aboriginal and non-Aboriginal populations. Exposures include: (**a**) Historical annual average maximum heatwave duration (days), 1990–2019; (**b**) Historical annual days exceeding 35 °C, 1990–2019; (**c**) Projected additional days exceeding 35 °C annually, 2020–2039; (**d**) Historical annual rainfall variability, 1990–2019; (**e**) Historical annual rainfall in millimetres (mm), 1990–2019; (**f**) Projected relative change in annual rainfall, 2020–2039. Triangle markers denote identified discrete Aboriginal communities. See Appendix A for a summary of descriptive statistics for selected climate exposure estimates on a continuous scale.

**Figure 3:**
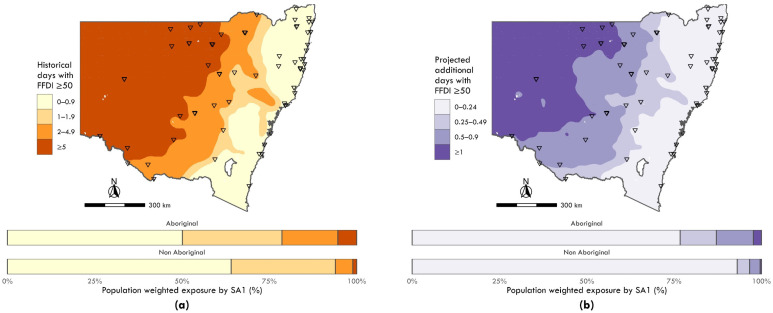
Maps of annual days with Macarthur Forest Fire Danger Index exceeding 50 (i.e., “severe” fire danger), with bar charts indicating relative exposure by category across Aboriginal and non-Aboriginal populations: (**a**) historical between 1990 and 2009; and (**b**) projected for 2020–2039. See Appendix A for a summary of descriptive statistics.

Figure 2 and Figure 3 show the levels of historical and projected climate exposure for heat, rainfall and fire danger in categories of increasing severity across NSW. The proportions of Aboriginal versus non-Aboriginal populations exposed to each category of climate exposure are provided in the histograms for each exposure map.

Figure 2a,b respectively shows the current impact of heatwaves and high temperatures (days exceeding 35 °C) across NSW, particularly affecting the north-west of the state. Figure 2c shows north-west NSW was also predicted to experience more than 10 additional days over 35 °C. The histograms associated with each figure show the disproportionate impact of these exposures on Aboriginal populations.

Figure 2d shows that rainfall variability was greatest in the west of the state, which also experiences the lowest annual rainfall (Figure 2e). The projected change in rainfall shown in Figure 2f was predicted to decrease in the southern parts of the state and increase across the north of the state, including coastal and inland areas. The histograms associated with Figure 2d,e shows that Aboriginal people disproportionately resided in areas of both lower and higher annual rainfall and experienced greater rainfall variability than the non-Aboriginal population of NSW. The histogram in Figure 2f shows that predicted changes in rainfall would disproportionately impact Aboriginal populations who would experience periods of both higher and lower rainfall over the next 20 years.

Figure 3a,b respectively shows the observed and predicted days of greater fire danger risk increased towards the west of the state. The histograms that show a higher proportion of the NSW Aboriginal populations historically resided in areas of greater fire danger risk compared to the non-Aboriginal population, and this was predicted to increase as the fire danger risk increases over the next 20 years.

### 3.2. Comparative Climate Exposure Levels by Index of Relative Socioeconomic Disadvantage (IRSD) between Aboriginal and Non-Aboriginal Populations

Sensitivity analyses revealed that Aboriginal people were at a disproportionately higher risk of exposure to all study climate parameters, regardless of socioeconomic status. In fact, for most exposures, Aboriginal people living in the highest IRSD quintile (i.e., the least disadvantaged) had higher odds of living in areas exposed to higher risk compared to non-Aboriginal populations in the same IRSD quintile. These results indicated that socioeconomic status alone did not account for the differential climate risk exposures for Aboriginal populations identified above. These results are displayed in Appendix A.

## 4. Discussion

Through the analysis of publicly available data and exposure mapping of climate hazards, this study confirmed what has been suspected anecdotally—that Aboriginal populations are disproportionately exposed to climate hazards, and this disparity is predicted to become more pronounced in the coming decades due to the projected impacts of climate change. The study quantified the different exposures for Aboriginal and non-Aboriginal populations in NSW, which include greater rainfall variability that can lead to more floods and droughts and higher temperatures and longer heatwaves that can result in greater risks of fire weather and bushfires. The Australian continent was romantically described by non-Aboriginal poet Dorothea Mackellar in her famous 1908 poem “My Country” as “a land of droughts and flooding rains” [33]. Anthropogenic changes in climate since this time have seen an amplification of these harsh extremes, and the health implications on Aboriginal populations, in particular, will depend on the effectiveness of the adaptation responses implemented today. The key issues and challenges faced by Aboriginal people under each study climate hazard are discussed below.

### 4.1. Increased Temperatures and Heatwaves

The analysis identified that a larger proportion of Aboriginal than non-Aboriginal people in NSW reside in areas that experienced greater numbers of hot days. These trends will continue as temperatures are predicted to increase with climate change.

Sensitivities to heat can be exacerbated by inadequate housing conditions, which is a key contributor to the health gap between Aboriginal and non-Aboriginal populations [34,35,36]. Housing issues are most prevalent in social housing, the dominant tenure type for Aboriginal communities [37,38]. Recent analysis of NSW housing condition data from 1998 to 2017 has indicated that housing conditions are unable to support basic healthy living practices (such as electrical safety, ablutions and food preparation) in the majority of Aboriginal community housing, and Aboriginal housing often lacks adequate thermal regulation, which puts older adults (particularly those with chronic disease) and children at risk of extreme temperature events [39,40]. Overcrowded conditions, together with poor housing amongst Aboriginal communities across Australia, have been recorded over many decades [38,40,41,42,43]. Overcrowding encourages the spread of infectious diseases, which can be intensified where only part of the house effectively regulates thermal comfort [44].

For NSW Aboriginal people already living in poor housing conditions, the projected impacts of climate change will add an additional level of complexity. As a key determinant of health and well-being, housing has the potential to either protect residents against or expose them to increased risk of climate-related harm [45].

Providing a cool living environment will be a key supportive factor in climate adaptability. Cooling housing can be achieved through a combination of passive design measures such as insulation, roof space ventilation or window shading and through active cooling measures such as air-conditioning or fans, which require ongoing running costs. A temperature control study in an Aboriginal community in north-western NSW in 2003 found the summer temperatures inside the houses exceeded 35 °C for up to 15 h of the day, including after 9 pm when children were trying to sleep [46]. Many houses had some form of active cooling but usually only in one room. As a result, families slept in the one cooled room at night, increasing the effects of crowding in the house. Passive cooling measures retrofitted to houses as part of the project reduced temperatures within the living space and improved the efficiency of mechanical cooling systems, but alone were unable to achieve thermal comfort [46]. Food security may also be affected by increased and prolonged temperatures. In NSW, Housing for Health data reported that 62% of Aboriginal homes surveyed between 1998 and 2017 had adequate refrigeration, and only 9% had adequate facilities to store, prepare and cook food [47]. The impact of inadequate housing design on Aboriginal health in NSW has been previously documented [40,46,48], and our mapping and projections show that this will likely be exacerbated by climate change.

Passive and active technical solutions exist to assist adaptive capacity to climate change, but any capital investment in social housing to cope with climate change needs to be supplemented by a robust and effective system of repair and maintenance. The NSW Housing for Health program identified that the overwhelming reason items failed in Aboriginal community housing was due to a lack of systematic maintenance (84%) and quality control (11%) [40].

Adaptation strategies must also be affordable so as not to burden social housing tenants already under financial stress with an additional economic demand beyond their capacity to pay [49]. Installation of active air cooling must also be supported by passive cooling measures to improve efficiency and minimise running costs. Adaptation strategies also need to consider impacts on utilities. High water consumption by evaporative cooling can impact water supplies [46], and increased use of air-conditioning over recent years has impacted the electricity grid in major cities, with substations overheating on days of high consumption [50,51,52]. Improvements in solar power technology over recent decades can reduce running costs and the impact on the electricity grid; however, these solutions require community engagement, robust systems of quality control and ongoing maintenance and monitoring so as not to provide an additional burden on communities.

### 4.2. Rainfall Variability and Risks of Flooding and Drought

A larger proportion of Aboriginal than non-Aboriginal people reside in areas that currently experience higher rainfall variabilities and risks of flooding and drought. The historical location of discrete Aboriginal communities near rivers will make those communities more susceptible to flooding and associated health issues. A survey conducted following the devastating floods of 2017 in Northern NSW showed that Aboriginal respondents had four times the odds of reporting flooded homes compared to non-Aboriginal respondents [53]. They were also more likely to report symptoms of anxiety, depression and post-traumatic stress disorder [53]. Other health risks associated with flood events are damages to sewage and water supply infrastructure leading to pathogen proliferation and water-borne diseases [36]. More extreme flooding in the same region in early 2022 is likely to lead to a cumulative impact from these climate risk factors.

Increased flooding events will impact communities along coastal and river systems, and the need for adequate flood mitigation strategies and disaster management preparedness will be crucial adaptation responses for these communities to protect against the loss of lives and assets. Increased flooding may also increase the risk to food and medication supply chains for regional and remote communities and transport logistics for agriculture, thus impacting food security and rural economies and increasing the need for support services in these communities.

Warmer temperatures and changed geographical patterns of intense rainfall or flooding, especially where “re-wetting” of an environment occurs, may promote breeding conditions for mosquitos and the transmission of vector-borne diseases [54]. Aboriginal people living in regional and remote areas can be disproportionately affected. The 2022 summer has seen the first incursion of Japanese encephalitis into south-eastern Australia [55,56].

Extremes of flooding and drought are also likely to have long-term impacts on agricultural production and local economies. Food insecurity may be exacerbated directly via crop damage or via scarcity-related food price increases [57]. The collapse of smaller local producers unable to survive prolonged droughts can lead to population decline in smaller regional centres, and the mental health impacts for residents of these towns should also be considered.

On average, a larger proportion of Aboriginal people than non-Aboriginal people resided in areas that experienced additional drought events in the past decade. Rivers are not just a source of water but also a source of food, recreation and connection and often hold the stories of the land integral to the maintenance and protection of Aboriginal cultures and practices. Reduced water flows during extended droughts will see more frequent drying up of river systems and increased algal blooms, affecting water supplies, diminishing fish and crayfish food sources and impacting swimming holes and other recreational activities.

Periods of extended drought can also affect the size and distribution of many native plants and animals that provide a traditional food source to many Aboriginal people and may represent cultural totems. Prolonged periods of drought are likely to reduce the availability of bush fruits, numbers of kangaroos and other animals, or the laying of emu eggs, for example, impacting food security. Collecting bush tucker not only supplements diet and provides an affordable food source, but it also reaffirms connection to Country.

With reduced water flows predicted during increasing drought conditions, water will become a scarcer commodity for rural communities dependent upon it for drinking, irrigation, agriculture and tourism. There may be engineering solutions that can assist with securing drinking water supplies in larger towns and communities in these regions, but few water utilities in regional and remote NSW have the financial capacity to upgrade systems without subsidy. Blending bore water with river water in periods of reduced river flow is common, but whilst this water meets potable guidelines, it may be far less palatable for drinking and bathing with higher levels of salts and other chemicals and a high alkaline texture. Reduced river water flows will also impact the ability to meet closing the gap targets for employment in regional areas where employment relies on agriculture and tourism.

In Australia, the Murray–Darling Basin catchment and tributaries cover 75% of NSW and are home to approximately 40 Aboriginal Nations or 15% of the total Aboriginal and Torres Strait Islander population [58]. To all Aboriginal groups, it is vitally important that the basin functions as a healthy, living river with natural flows and cycles. However, the basin also provides drinking water for over 3 million people and supports 45% of Australia’s agricultural output, resulting in water over-extraction and reversal of cultural flows [58]. For improved sustainability, Aboriginal cultural and spiritual values should be considered in the allocation of water alongside market value [59,60]. There is a need to engage now with Aboriginal communities all along the river systems in developing river water management solutions that consider the predicted impacts of climate change.

### 4.3. Fire Danger

A higher proportion of Aboriginal people reside in areas exposed to higher fire danger days than non-Aboriginal people. During the summer bushfires of 2019/2020, 7% or 5 million hectares of land on the eastern seaboard were burnt [61]. Of the total Aboriginal population living in NSW and Victoria, greater than 25% lived in fire-affected areas, of which 36% were Aboriginal children [62]. Smoke inhalation, as an indirect health impact of bushfires, is particularly harmful to children’s respiratory health [63]. Bushfires also contribute to trauma-related anxiety and loss of homes, traditional resources and cultural sites. The Aboriginal experience of a bushfire disaster is distinctively intimate and akin to the burning up of memories, sacred places and “someone far older and wiser” [64]. Increased bushfires in water supply catchments will also impact drinking water quality management.

Climate change will likely increase the frequency and intensity of bushfires, and more Aboriginal people will be disproportionately affected. Given that Aboriginal people hold valuable knowledge on restorative traditional fire practices and significant legal rights and cultural heritage in lands and waters in fire-prone areas, it is important to recognise their place in the planning and implementation of bushfire disaster response strategies and policies, as well as cultural heritage protection and Indigenous fire management [64].

### 4.4. Culturally Centred Approach toward Climate Adaptation

**Figure 4:**
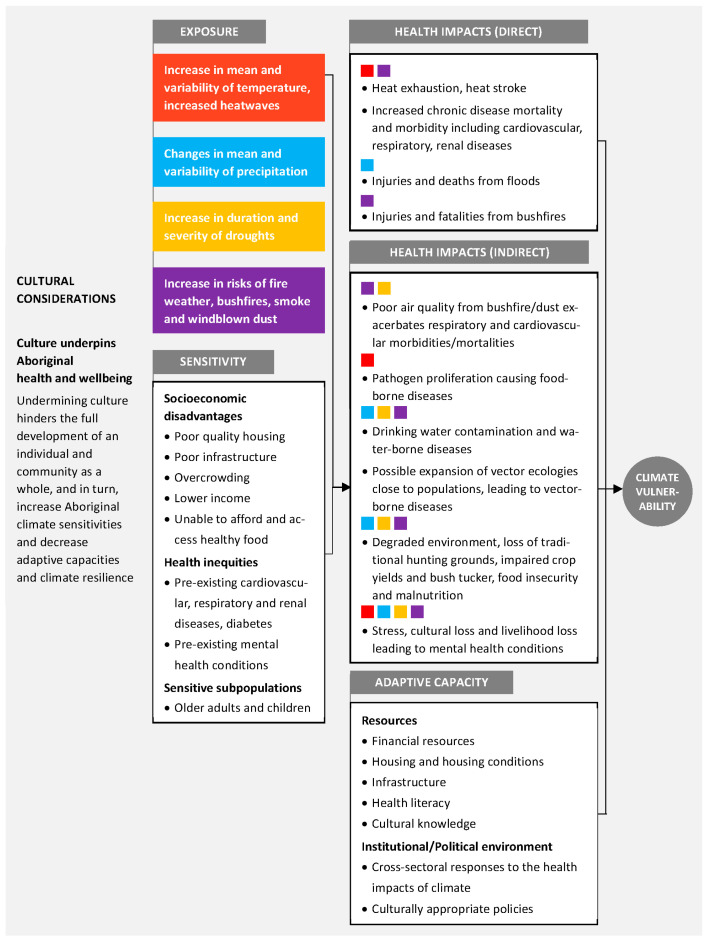
Summary of the components of climate vulnerability, where exposure to climate-related hazards and sensitivity factors contribute to the susceptibility of Aboriginal people to a range of direct and indirect health outcomes. Capacity to manage the influence of exposure and sensitivities to climate-related hazards can reduce the net vulnerability to climate change. (Adapted from [67]). Underpinning the components of climate vulnerability are the complex and important cultural determinants [68] of Aboriginal health and well-being.

Figure 4 summarises factors that contribute to the experience of climate vulnerability for Aboriginal people in the context of exposure, sensitivity and adaptive capacities. Living in areas prone to extreme heat, fire weather, floods and droughts under climate change will impact the health and well-being of Aboriginal populations directly and indirectly. These health impacts are exacerbated by existing ill-health amongst Aboriginal communities and compounded by factors such as poor housing and food insecurity. Capacities to reduce climate exposures and sensitivities will depend on effective technologies, adequate financial resources and supportive institutions and governance [65]. Informed by NSW Aboriginal agency stakeholders working in the Aboriginal community and health sectors, it became evident that undermining Aboriginal cultures impacts adversely Aboriginal health and well-being, which will, in turn, increase climate vulnerability. Cultural determinants add an additional layer to the framework in its application to NSW Aboriginal communities. This is consistent with previous findings described in the literature that have identified the impact of climate change on Indigenous cultures in Australia and internationally [66] and our sensitivity analysis, which suggested a higher exposure to climate-related hazards for Aboriginal populations in NSW over and above socioeconomic disadvantage compared to the broader, non-Aboriginal population.

### 4.5. Policy Implications

The recognition that culture is an underlying determinant of good health is in line with the national “Closing the Gap” strategy, which advocates for an Aboriginal- and Torres Strait Islander-driven approach to health policy and program reform [68]. This strength-based approach is key to building resilience to the impacts of climate on Aboriginal health, and action is needed now to develop effective Aboriginal-led adaptation responses to the current and future impacts of climate on health.

The increased exposures identified in this analysis, along with climate sensitivities already experienced by Aboriginal populations in NSW, such as socioeconomic and health inequalities, indicate that the potential health impacts of climate change are even more significant for those populations than it is for the non-Aboriginal populations in NSW. This combination of inequities in exposure, sensitivity and adaptive capacity to climate-related hazards in Aboriginal populations can be described as both climate and racial injustice [69,70], particularly as susceptibility to these is linked to structural inequalities in society. These injustices identified within Australia are analogous to those seen between countries whereby climate risks are inequitably borne by post-colonial lower-income countries and other First Nations communities [66,71,72]. The Australian government has set 17 “Closing the Gap” targets aimed at improving socioeconomic outcomes for Aboriginal and Torres Strait Islander people, including health and well-being, education, employment, justice, safety, housing, land and waters, languages and digital inclusion outcomes [73]. Climate change is likely to widen the inequities that currently exist in relation to many of these factors.

Undermining Aboriginal cultures in responding to climate change not only increases sensitivity but decreases adaptive capacity. As climate change progresses, the capacity to adapt to these greater potential health impacts is further compounded by several non-climatic stressors already experienced by Aboriginal populations related to socioeconomics (such as financial inequity, health literacy and the condition of housing and infrastructure), and factors in the institutional and political environments (such as government policy related to allopathic approaches to health service delivery, management of social housing and infrastructure, or water allocations). In addition, these stressors exist within historical experiences of disempowerment of Aboriginal people in Australia, such as colonisation and land dispossession, experiences that have been shared by other First Nations people globally [66,71,72]. Conversely, policies that seek to address the underlying determinants of health, such as reducing financial inequity, improving health literacy and housing and further empowering Aboriginal communities, may improve adaptive capacity to climate-related health risks.

Connection to Country was identified by Aboriginal agency stakeholders as a key component of Aboriginal identity, with many NSW Aboriginal people living where they do because of cultural and ancestral connections to the land and waters of those places going back for tens of thousands of years. Whilst changes to the climate will impact many of those places with more extreme, harsher living conditions predicted and increased fire danger, simply moving “off Country” would have additional cultural, health and well-being implications [74].

What is lesser understood is the role traditional Aboriginal knowledges play in climate adaptation. Aboriginal people have inhabited the Australian continent for tens of thousands of years, and learnings from traditional knowledges could benefit the wider population [72]. Traditional, cooler burning practices, for example, are being incorporated into fire management in some areas, and researchers are working with traditional owners to understand these practices and their benefits for managing fires now and into the future [75,76,77].

The NSW government recognises the potential impact climate change may have on government assets and service delivery and their ability to meet policy objectives and is in the process of developing a NSW climate change adaptation strategy [78]. It has identified benefits for the state from the economic and employment opportunities adaptation offers and claims the strategy will “give NSW families and communities confidence the challenges posed by climate change can be solved by improving–not eroding their prosperity”. How this strategy will engage the Aboriginal people of NSW has not yet been defined, but history would indicate there is a very tangible risk that Aboriginal communities may be left out of these discussions, with unintended consequences exacerbating health inequities. Now is the time to be engaging with Aboriginal communities to ensure Aboriginal knowledges are incorporated and climate change adaptation offers opportunities for all.

The US Public Health Institute’s statement on climate change describes the concept of “Health, Equity and Climate in All Policies” and calls on federal agencies to consider the impact of their decisions on climate, health and equity [79]. NSW Health requires an Aboriginal Health Impact Statement to be applied to all health policies, programs and strategies to ensure the health and well-being of Aboriginal people have been adequately considered [80]. Given many environmental factors that impact health sit within the responsibility of other agencies such as housing or infrastructure, a similar systematic approach should be applied more broadly to all agencies at all levels of government to ensure appropriate Aboriginal community engagement and consideration of climate, health and equity impacts for all government policies, planning and developments.

The timely implementation of strategies is critical. Aboriginal people in NSW were identified early in the COVID-19 pandemic response as a sensitive population to the disease given existing health co-morbidities, housing conditions and socioeconomic factors. However, until there was an outbreak in the Aboriginal community, limited resources were directed towards this population, compromising an effective response. The same approach cannot afford to be taken to address climate change.

### 4.6. Strengths and Limitations

All population data used in this study was from 2016 populations, both the URPs at the SA1 level and overall populations from the Australian population grid. This means that projection estimates assume that the proportions of Aboriginal vs. non-Aboriginal people at the SA1 level will remain static. While changes in these populations are to be expected [81], especially into the future, overall proportions are unlikely to change substantially. Indeed, analysis of 2006, 2011 and 2016 censuses revealed only small changes in the proportions of Aboriginal vs. non-Aboriginal populations at the SA1 level, with very little internal migration observed across rural and regional areas. As such, these estimates should be indicative of the population proportions in the near future.

The preliminary consultations in this paper were far from exhaustive, and for a population that stands to be disproportionately impacted by climate change, further work needs to be done to engage the Aboriginal community in developing and implementing appropriate adaptation strategies.

While mitigation is recognised as an essential component of effective adaptation, this paper focuses on climate change adaptation. Further development of this work should include a combined focus on mutually reinforcing mitigation and adaptation actions to reduce the burden of Aboriginal ill health, boost community resilience and lessen poverty and inequity.

## 5. Conclusions

This analysis demonstrates that the Aboriginal population of NSW are currently disproportionally exposed to extreme climate events, and this disproportionate exposure will increase in the future with predicted changes in climate. Pre-existing health sensitivities (such as chronic diseases and psychosocial distress) and factors limiting adaptive capacity (such as socioeconomic disadvantage and political environments) already put Aboriginal populations in NSW at greater risk of the impacts of climate change, and this will continue unless significant challenges are addressed.

Based on guidance, stories and experiences from the Aboriginal stakeholders and authors involved in the project, the following recommendations are proposed to assist local communities and all levels of government in guiding the development and implementation of adaptation policies and strategies underpinned by Aboriginal cultural considerations.

1. Opportunities for communities

Effective engagement with Aboriginal communities, supported by active investment, is urgently needed to identify and maximise the opportunities climate adaptation may offer to improve health and socioeconomic equity for communities.

2. Adaptation Strategies

Climate change needs to be a key consideration in all future planning by policymakers and program implementers across all tiers of government. A “Climate Change, Health and Equity Impact Statement” should accompany any future policies, strategies or planning developments at all levels of government, identifying impacts (both positive and negative) on climate change, health and equity, including mitigation and adaptation strategies, particularly on populations vulnerable to climate change. This should be a consideration of all policies, standards and guidelines, from the design of housing through to the strategic energy needs of the nation.

3. Implementation and management

Climate adaptation strategies are needed now that ensure responses to health and social service preparedness, including housing, water supplies, infrastructure and food security are effectively implemented and managed. Quality control and maintenance systems for these services, particularly social housing, must be adequately supported and resourced to be implemented effectively.

4. Communication and cultural adaptation strategies

The true impact of climate on NSW Aboriginal populations and cultural practices is not clearly understood. A better understanding of current health impacts and adaptation responses among Aboriginal populations and communities in NSW is required to develop culturally appropriate adaptation strategies and resilient communities in the face of climate change. Further quantitative and qualitative participatory-based research to identify cultural impacts of climate change on health and explore adaptive responses, including those based on Aboriginal knowledges, should be supported.

## Figures and Tables

**Table 1:** Annual climate exposure estimates by Aboriginal versus non-Aboriginal URPs, population weighted at the Statistical Area 1 level for historical (1990–2019) and projected (2020–2039) periods.

Climate Exposure	Exposure Category	Aboriginal URP ^i^		Non-Aboriginal URP ^i^		Binary Risk Category	Higher-Risk Exposed Population (%)		Odds Ratio
		*n*	*%*	*n*	*%*		Aboriginal	Non-Aboriginal	
Historical annual average maximum heatwave days ^ii^	0–4	12,882	6.2%	712,057	10.0%	Lower			3.79 [3.75–3.83] **
	5	101,001	48.9%	5,109,987	71.6%	Lower			
	6	38,193	18.5%	696,651	9.8%	Lower			
	7	37,112	18.0%	496,711	7.0%	Higher	26.4%	8.7%	
	>7	17,514	8.5%	121,239	1.7%	Higher			
Historical annual days above 35 °C	<5	61,112	29.3%	1,768,115	24.5%	Lower			4.34 [4.29–4.39] **
	5–9.9	64,413	30.9%	4,122,137	57.2%	Lower			
	10–19.9	42,504	20.4%	932,265	12.9%	Lower			
	20–49.9	34,536	16.6%	360,230	5.0%	Higher	19.5%	5.3%	
	≥50	6101	2.9%	19,784	0.3%	Higher			
Projected additional days above 35 °C	<2	59,097	28.0%	1,842,597	25.4%	Lower			3.45 [3.42–3.48] **
	2–4.9	84,608	40.1%	4,536,671	62.6%	Lower			
	5–9.9	45,609	21.6%	760,717	10.5%	Higher	31.8%	11.9%	
	≥10	21,470	10.2%	102,944	1.4%	Higher			
Historical annual rainfall variability	Low	25,477	12.2%	1,920,044	26.7%	Lower			4.67 [4.61–4.73] **
	Low to moderate	155,450	74.5%	5,053,399	70.2%	Lower			
	Moderate	23,201	11.1%	204,412	2.8%	Higher	13.3%	3.2%	
	Moderate to high	2864	1.4%	7912	0.1%	Higher			
	High	1674	0.8%	16,764	0.2%	Higher			
Historical annual average rainfall (mm)	≤400	8138	3.9%	129,981	1.8%	Higher	35.6%	16.6%	2.77 [2.75–2.80] **
	400–799	66,068	31.7%	1,065,592	14.8%	Higher			
	800–1199	110,212	52.8%	5,243,879	72.8%	Lower			
	≥1200	24,248	11.6%	763,079	10.6%	Lower			
Projected change in annual rainfall (%)	<−1%	23,675	11.2%	412,395	5.7%	Higher	23.3%	12.4%	2.14 [2.12–2.16] **
	−0.01	25,384	12.0%	487,009	6.7%	Higher			
	0–0.9%	17,531	8.3%	491,761	6.8%	Lower			
	1–1.9%	71,977	34.1%	3,863,646	53.3%	Lower			
	≥2%	72,211	34.3%	1,987,405	27.4%	Lower			
Historical annual days with FFDI ≥ 50 ^iii^	0–0.9	109,645	52.8%	4,779,751	66.9%	Lower			4.26 [4.21–4.31] **
	1–1.9	55,888	26.9%	1,962,018	27.5%	Lower			
	2–4.9	31,580	15.2%	329,493	4.6%	Higher	20.4%	5.7%	
	≥5	10,731	5.2%	74,910	1.0%	Higher			
Projected additional annual days FFDI ≥50	0–0.24	161,178	77.8%	6,659,154	93.5%	Lower			4.35 [4.29–4.41] **
	0.25–0.49	20,318	9.8%	236,287	3.3%	Lower			
	0.5–0.9	20,991	10.1%	198,057	2.8%	Higher	12.4%	3.1%	
	≥1	4599	2.2%	25,478	0.4%	Higher			
Change in drought-affected months (1990–2006 vs. 2007–2020)	≤−10%	40,473	20.1%	2,409,632	35.8%	Lower			2.35 [2.33–2.37] **
	−9.9–−5%	58,985	29.3%	2,436,940	36.2%	Lower			
	−4.9–−2.5%	19,621	9.7%	435,573	6.5%	Lower			
	−2.4–0%	17,825	8.9%	333,644	5.0%	Lower			
	0.01–5%	39,283	19.5%	753,751	11.2%	Higher	32.0%	16.7%	
	>5%	25,159	12.5%	370,407	5.5%	Higher			

Notes: (i) URP = usual resident populations from the 2016 Australian Bureau of Statistics Census of Population and Housing; ** *p* < 0.001; (ii) Annual average maximum heatwave days refer to the average longest heatwave duration (in days), where each day’s rolling three-day mean temperature both exceeds the 95th percentile of historical temperatures and the recent 30-day average for that region; Historical period is from 1990 to 2019 except (iii) Historical FFDI where the time period is 1990–2009. Projected period is from 2020 to 2039.

**Figure A1:**
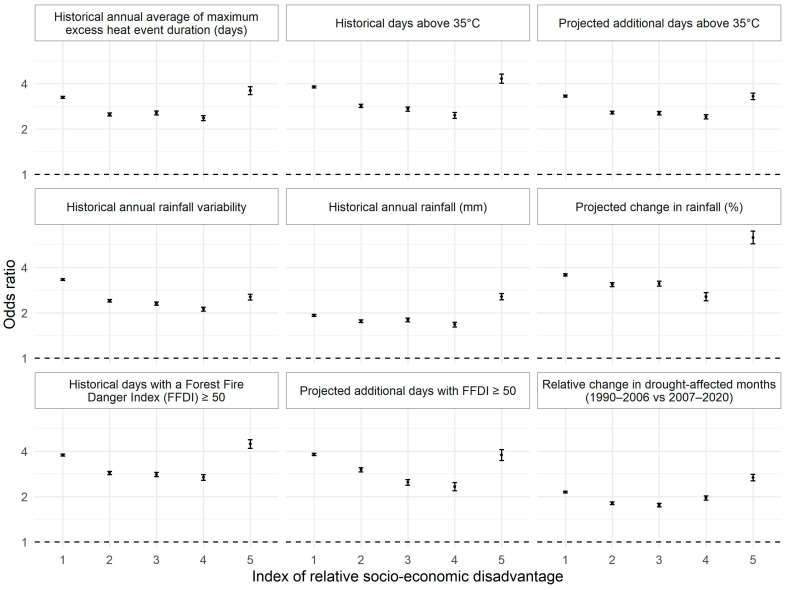
Climate exposure estimates by Aboriginal versus non-Aboriginal usual resident populations stratified by the Index of Relative Socioeconomic Disadvantage (IRSD).

## Data Availability

Publicly available data sources are referenced in the paper.

## Appendix A

Table A1 presents a summary of descriptive statistics for selected climate exposure estimates on a continuous scale. Table A2 and Figure A1 present the results of the sensitivity analysis. In these analyses, odds ratios of high-risk environmental exposure are recalculated and stratified by the Australian Bureau of Statistics Index of Relative Socioeconomic Disadvantage (IRSD) quintiles. An IRSD of 1 represents the level of greatest disadvantage, and 5 the lowest level of disadvantage.

**Table A1:** Descriptive statistics for selected climate exposure estimates on a continuous scale.

Exposure	Mean	Standard Deviation%	Minimum	Median	25th Percentile	75th Percentile	Maximum
Historical annual maximum heatwave duration (days)	4.50	1.02	0.00	4.08	4.25	4.56	8.02
Historical annual days over 35 °C (days)	8.51	8.19	0.00	4.92	6.80	9.35	84.17
Projected additional days over 35 °C (days)	3.35	2.22	0.00	1.93	2.87	4.05	15.22
Historical average annual rainfall (mm)	968.43	255.24	0.00	834.57	993.16	1132.05	1779.36
Projected change in annual rainfall (%)	1.36	1.18	−4.10	1.19	1.66	2.01	3.97
Historical annual days with FFDI ≥ 50 (days)	0.94	1.15	0.00	0.42	0.72	1.13	16.05
Projected additional annual days with FFDI ≥ 50 (days)	0.09	0.17	−0.05	0.01	0.04	0.08	1.71

**Table A2:** Climate exposure estimates by Aboriginal versus non-Aboriginal usual resident populations stratified by the Index of Relative Socioeconomic Disadvantage (IRSD). Small cell counts for either population have been aggregated.

Climate Exposure	IRSD Quintile	Exposure Category	Aboriginal URP		Non-Aboriginal URP		Binary Risk Category	High-Risk Exposure Population (%)		Odds Ratio
			n	%	n	%		Aboriginal	Non-Aboriginal	
Historical annual average maximum heatwave duration (day)	1	0–4	3658	4.0%	67,183	4.5%	Lower			3.24 [3.19–3.28]
		5	36,193	39.3%	1,006,257	67.0%	Lower			
		6	19,776	21.4%	210,839	14.0%	Lower			
		7	22,740	24.7%	180,293	12.0%	Higher	35.3%	14.4%	
		>7	9831	10.7%	36,434	2.4%	Higher			
	2	0–4	2990	6.3%	90,031	6.4%	Lower			2.45 [2.40–2.50]
		5	24,199	51.3%	978,283	69.5%	Lower			
		6	9025	19.1%	184,468	13.1%	Lower			
		7	7033	14.9%	125,239	8.9%	Higher	23.2%	11.0%	
		>7	3881	8.2%	28,956	2.1%	Higher			
	3	0–4	2150	7.2%	93,278	7.5%	Lower			2.54 [2.46–2.61]
		5	17,155	57.1%	908,316	72.6%	Lower			
		6	4731	15.7%	136,558	10.9%	Lower			
		7	4054	13.5%	89,254	7.1%	Higher	20.0%	9.0%	
		>7	1965	6.5%	23,086	1.8%	Higher			
	4	0–4	1801	8.7%	133,554	10.9%	Lower			2.40 [2.31–2.49]
		5	13,301	64.3%	916,272	74.5%	Lower			
		6	2364	11.4%	91,407	7.4%	Lower			
		7	2066	10.0%	67,511	5.5%	Higher	15.6%	7.1%	
		>7	1161	5.6%	20,335	1.7%	Higher			
	5	0–4	1957	14.9%	320,458	18.6%	Lower			3.82 [3.60–4.06]
		5	8736	66.5%	1283,494	74.7%	Lower			
		6	1232	9.4%	69,650	4.1%	Lower			
		7	548	4.2%	32,345	1.9%	Higher	9.3%	2.6%	
		>7	670	5.1%	12,362	0.7%	Higher			
Historical annual days above 35 °C	1	<5	25,453	27.4%	349,476	23.1%	Lower			3.79 [3.73–3.85]
		5–9.9	22,351	24.1%	792,040	52.4%	Lower			
		10–19.9	20,087	21.6%	235,915	15.6%	Lower			
		20–49.9	20,533	22.1%	125,376	8.3%	Higher	26.8%	8.8%	
		≥50	4367	4.7%	7809	0.5%	Higher			
	2	<5	16,297	34.2%	402,935	28.3%	Lower			2.79 [2.72–2.86]
		5–9.9	14,152	29.7%	705,818	49.6%	Lower			
		10–19.9	9375	19.7%	221,492	15.6%	Lower			
		20–49.9	6625	13.9%	87,296	6.1%	Higher	16.4%	6.6%	
		≥50	1187	2.5%	6169	0.4%	Higher			
	3	<5	9335	30.6%	307,539	24.3%	Lower			2.69 [2.60–2.78]
		5–9.9	10,624	34.9%	712,058	56.2%	Lower			
		10–19.9	6093	20.0%	172,158	13.6%	Lower			
		20–49.9	4094	13.4%	72,327	5.7%	Higher	14.5%	5.9%	
		≥50	319	1.0%	2808	0.2%	Higher			
	4	<5	5657	26.9%	293,237	23.5%	Lower			2.50 [2.39–2.62]
		5–9.9	8789	41.8%	740,170	59.4%	Lower			
		10–19.9	4531	21.5%	160,069	12.9%	Lower			
		20–49.9	1897	9.0%	48,930	3.9%	Higher	9.8%	4.2%	
		≥50	158	0.8%	2766	0.2%	Higher			
	5	<5	3316	25.0%	405,768	23.5%	Lower			4.57 [4.26–4.91]
		5–9.9	7351	55.4%	1,154,813	66.9%	Lower			
		10–19.9	1748	13.2%	139,522	8.1%	Lower			
		20–49.9	832	6.3%	25,023	1.5%	Higher	6.4%	1.5%	
		≥50	10	0.1%	191	0.0%	Higher			
Projected additional days above 35 °C	1	<2	24,079	25.7%	365,971	24.0%	Lower			3.26 [3.21–3.30]
		2–4.9	32,962	35.2%	907,961	59.5%	Lower			
		5–9.9	22,451	24.0%	211,402	13.9%	Higher	39.1%	16.5%	
		≥10	14,164	15.1%	39,770	2.6%	Higher			
	2	<2	16,131	33.4%	417,526	29.0%	Lower			2.48 [2.43–2.53]
		2–4.9	18,751	38.8%	827,918	57.6%	Lower			
		5–9.9	9344	19.3%	168,441	11.7%	Higher	27.8%	13.4%	
		≥10	4074	8.4%	24,698	1.7%	Higher			
	3	<2	8952	28.9%	325,981	25.5%	Lower			2.47 [2.41–2.54]
		2–4.9	13,308	42.9%	779,115	60.9%	Lower			
		5–9.9	6922	22.3%	156,139	12.2%	Higher	28.2%	13.7%	
		≥10	1809	5.8%	19,137	1.5%	Higher			
	4	<2	4763	24%	260,312	23%	Lower			2.42 [2.35–2.50]
		2–4.9	9484	48%	727,159	64%	Lower			
		5–9.9	4516	23%	141,911	12%	Higher	25.6%	12.5%	
		≥10	891	5%	13,413	1%	Higher			
	5	<2	3473	26.2%	403,194	23.4%	Lower			3.46 [3.29–3.62]
		2–4.9	7749	58.4%	1,231,131	71.6%	Lower			
		5–9.9	1685	12.7%	80,360	4.7%	Higher	15.4%	5.0%	
		≥10	360	2.7%	5839	0.3%	Higher			
Historical annual rainfall variability	1	Low	8486	9.1%	177,202	11.7%	Lower			3.55 [3.49–3.62]
		Low to moderate	66,796	72.0%	1,240,577	82.1%	Lower			
		Moderate	14,004	15.1%	77,837	5.2%	Higher	18.9%	6.1%	
		Moderate to high	2045	2.2%	2508	0.2%	Higher			
		High	1460	1.6%	12,492	0.8%	Higher			
	2	Low	5181	10.9%	289,093	20.3%	Lower			3.05 [2.96–3.14]
		Low to moderate	37,225	78.1%	1,079,304	75.8%	Lower			
		Moderate	4468	9.4%	49,810	3.5%	Higher			
		Moderate to high	646	1.4%	3447	0.2%	Higher	11.0%	3.9%	
		High	116	0.2%	2056	0.1%	Higher			
	3	Low	4082	13.4%	337,452	26.6%	Lower			3.08 [2.97–3.21]
		Low to moderate	23,270	76.4%	884,359	69.8%	Lower			
		Moderate	2903	9.5%	42,098	3.3%	Higher	10.2%	3.6%	
		Moderate to high	120	0.4%	1189	0.1%	Higher			
		High	90	0.3%	1792	0.1%	Higher			
	4	Low	3404	16.2%	362,383	29.1%	Lower			2.60 [2.45–2.76]
		Low to moderate	16,482	78.4%	855,795	68.7%	Lower			
		Moderate	1085	5.2%	25,904	2.1%	Higher	5.4%	2.2%	
		Moderate to high	53	0.3%	727	0.1%	Higher			
		High	8	0.0%	363	0.0%	Higher			
	5	Low	3938	29.7%	743,085	43.1%	Lower			6.70 [6.08–7.39]
		Low to moderate	8882	67.0%	973,498	56.4%	Lower			
		Moderate	437	3.3%	8673	0.5%	Higher	3.3%	0.5%	
		Moderate to high	0	0.0%	0	0.0%	Higher			
		High	0	0.0%	61	0.0%	Higher			
Historical annual average rainfall (mm)	1	≤400	5377	5.8%	33,291	2.2%	Higher	42.0%	18.3%	3.24 [3.20–3.29]
		400–799	33,630	36.2%	242,707	16.1%	Higher			
		800–1199	44,544	48.0%	1,094,131	72.4%	Lower			
		≥1200	9240	10.0%	140,487	9.3%	Lower			
	2	≤400	1460	3.1%	22,859	1.6%	Higher	33.4%	18.1%	2.26 [2.22–2.30]
		400–799	14,431	30.3%	235,298	16.5%	Higher			
		800–1199	25,028	52.5%	998,470	70.1%	Lower			
		≥1200	6717	14.1%	167,083	11.7%	Lower			
	3	≤400	594	1.9%	15,759	1.2%	Higher	29.4%	16.0%	2.18 [2.13–2.24]
		400–799	8365	27.5%	187,141	14.8%	Higher			
		800–1199	17,321	56.9%	924,890	73.0%	Lower			
		≥1200	4185	13.7%	139,100	11.0%	Lower			
	4	≤400	286	1.4%	17,820	1.4%	Higher	29.0%	16.7%	2.03 [1.97–2.09]
		400–799	5815	27.6%	190,745	15.3%	Higher			
		800–1199	12,465	59.3%	902,392	72.5%	Lower			
		≥1200	2466	11.7%	134,215	10.8%	Lower			
	5	≤400	213	1.6%	40,008	2.3%	Higher	24.5%	14.1%	1.98 [1.90–2.06]
		400–799	3033	22.9%	202,899	11.8%	Higher			
		800–1199	8483	64.0%	1,301,490	75.4%	Lower			
		≥1200	1528	11.5%	180,920	10.5%	Lower			
Projected change in annual rainfall (%)	1	<−1%	11,296	12.1%	108,364	7.1%	Higher	25.4%	15.5%	1.86 [1.83–1.89]
		−1–0%	12,524	13.4%	128,238	8.4%	Higher			
		0–0.9%	8824	9.4%	102,000	6.7%	Lower			
		1–1.9%	25,290	27.0%	825,439	54.1%	Lower			
		≥2%	35,722	38.1%	361,063	23.7%	Lower			
	2	<−1%	5593	11.6%	103,775	7.2%	Higher	24.3%	15.9%	1.69 [1.65–1.73]
		−1–0%	6122	12.7%	125,318	8.7%	Higher			
		0–0.9%	3458	7.2%	103,196	7.2%	Lower			
		1–1.9%	17,570	36.4%	761,097	52.9%	Lower			
		≥2%	15,557	32.2%	345,197	24.0%	Lower			
	3	<−1%	3217	10.4%	74,245	5.8%	Higher	20.8%	13.0%	1.77 [1.72–1.82]
		−1–0%	3238	10.4%	91,787	7.2%	Higher			
		0–0.9%	2541	8.2%	97,250	7.6%	Lower			
		1–1.9%	12,187	39.3%	699,735	54.7%	Lower			
		≥2%	9808	31.6%	317,355	24.8%	Lower			
	4	<−1%	1866	8.8%	64,896	5.2%	Higher	18.6%	11.7%	1.71 [1.65–1.78]
		−1–0%	2046	9.7%	81,399	6.5%	Higher			
		0–0.9%	1612	7.6%	89,818	7.2%	Lower			
		1–1.9%	9234	43.8%	651,410	52.2%	Lower			
		≥2%	6327	30.0%	359,622	28.8%	Lower			
	5	<−1%	1211	9.1%	59,157	3.4%	Higher	16.4%	6.8%	2.70 [2.57–2.82
		−1–0%	962	7.3%	57,422	3.3%	Higher			
		0–0.9%	949	7.2%	98,508	5.7%	Lower			
		1–1.9%	6715	50.6%	908,782	52.8%	Lower			
		≥2%	3424	25.8%	596,231	34.7%	Lower			
Historical annual days with a Forest Fire Danger Index (FFDI) ≥ 50	1	0–0.9	42,363	45.8%	815,518	54.2%	Lower			3.76 [3.70–3.82]
		1–1.9	25,227	27.3%	555,757	36.9%	Lower			
		2–4.9	17,140	18.5%	100,019	6.6%	Higher	26.9%	8.9%	
		≥5	7769	8.4%	34,516	2.3%	Higher			
	2	0–0.9	27,506	58.0%	924,217	65.2%	Lower			2.81 [2.75–2.88]
		1–1.9	11,242	23.7%	388,921	27.4%	Lower			
		2–4.9	7040	14.8%	87,514	6.2%	Higher	18.3%	7.4%	
		≥5	1652	3.5%	17,233	1.2%	Higher			
	3	0–0.9	17,027	56.1%	826,286	65.7%	Lower			2.76 [2.67–2.85]
		1–1.9	8645	28.5%	352,969	28.1%	Lower			
		2–4.9	3898	12.9%	62,477	5.0%	Higher	15.4%	6.2%	
		≥5	763	2.5%	15,048	1.2%	Higher			
	4	0–0.9	11,881	56.6%	866,053	70.0%	Lower			2.71 [2.60–2.83]
		1–1.9	6629	31.6%	313,273	25.3%	Lower			
		2–4.9	2184	10.4%	50,300	4.1%	Higher	11.7%	4.7%	
		≥5	279	1.3%	7613	0.6%	Higher			
	5	0–0.9	8726	66.5%	1,324,281	78.0%	Lower			4.71 [4.40–5.03]
		1–1.9	3417	26.1%	345,003	20.3%	Lower			
		2–4.9	968	7.4%	27,970	1.6%	Higher	7.4%	1.7%	
		≥5	3	0.0%	399	0.0%	Higher			
Projected additional annual days with FFDI ≥ 50	1	0–0.24	65,220	70.7%	1,347,359	89.7%	Lower			3.80 [3.73–3.87]
		0.25–0.49	11,024	11.9%	76,160	5.1%	Lower			
		0.5–0.9	12,321	13.3%	61,945	4.1%	Higher	17.4%	5.2%	
		≥1	3731	4.0%	16,917	1.1%	Higher			
	2	0–0.24	37,683	79.8%	1,286,023	91.4%	Lower			2.99 [2.90–3.08]
		0.25–0.49	4589	9.7%	68,509	4.9%	Lower			
		0.5–0.9	4403	9.3%	48,374	3.4%	Higher	10.4%	3.8%	
		≥1	523	1.1%	4409	0.3%	Higher			
	3	0–0.24	25,169	83.2%	1,167,109	93.1%	Lower			2.46 [2.35–2.57]
		0.25–0.49	2748	9.1%	45,934	3.7%	Lower			
		0.5–0.9	2139	7.1%	38,021	3.0%	Higher	7.7%	3.3%	
		≥1	193	0.6%	3204	0.3%	Higher			
	4	0–0.24	18,091	87.1%	1,159,839	94.6%	Lower			2.36 [2.22–2.51]
		0.25–0.49	1538	7.4%	37,218	3.0%	Lower			
		0.5–0.9	1112	5.4%	28,621	2.3%	Higher	5.5%	2.4%	
		≥1	29	0.1%	823	0.1%	Higher			
	5	0–0.24	12,145	92.8%	1,669,391	98.3%	Lower			3.99 [3.67–4.34]
		0.25–0.49	346	2.6%	8289	0.5%	Lower			
		0.5–0.9	597	4.6%	20,020	1.2%	Higher	4.6%	1.2%	
		≥1	0	0.0%	61	0.0%	Higher			
Relative change in drought-affected months (1990–2006 vs. 2007–2020)	1	≤−10%	13,856	15.3%	344,184	23.3%	Lower			2.12 [2.09–2.15]
		−9.9–−5%	25,938	28.6%	609,131	41.2%	Lower			
		−4.9–−2.5%	9507	10.5%	135,713	9.2%	Lower			
		−2.4–0%	8878	9.8%	80,167	5.4%	Lower			
		0.01%–5%	17,490	19.3%	178,228	12.1%	Higher	35.9%	20.9%	
		>5%	15,134	16.7%	130,805	8.8%	Higher			
	2	≤10%	8460	18.5%	418,629	30.7%	Lower			1.76 [1.72–1.79]
		−9.9–−5%	13,802	30.2%	452,136	33.1%	Lower			
		−4.9%–−2.5%	4331	9.5%	109,654	8.0%	Lower			
		−2.4–0%	3622	7.9%	77,300	5.7%	Lower			
		0.01–5%	10,035	22.0%	202,484	14.8%	Higher	33.9%	22.5%	
		>5%	5427	11.9%	105,395	7.7%	Higher			
	3	≤−10%	6724	23.1%	459,306	38.2%	Lower			1.70 [1.66–1.75]
		−9.9–−5%	8323	28.6%	365,938	30.4%	Lower			
		−4.9–−2.5%	3383	11.6%	87,006	7.2%	Lower			
		−2.4–0%	2549	8.8%	68,078	5.7%	Lower			
		0.01–5%	5718	19.7%	153,570	12.8%	Higher	27.9%	18.5%	
		>5%	2401	8.3%	69,538	5.8%	Higher			
	4	≤−10%	6010	29.9%	526,829	45.0%	Lower			1.89 [1.83–1.96]
		−9.9–−5%	5613	27.9%	335,048	28.6%	Lower			
		−4.9–−2.5%	1503	7.5%	61,476	5.3%	Lower			
		−2.4–0%	1847	9.2%	66,667	5.7%	Lower			
		0.01–5%	3834	19.0%	134,397	11.5%	Higher	25.6%	15.4%	
		>5%	1324	6.6%	45,693	3.9%	Higher			
	5	≤−10%	4192	34.4%	645,501	43.2%	Lower			2.65 [2.53–2.79]
		−9.9–−5%	4677	38.4%	669,733	44.8%	Lower			
		−4.9–−2.5%	697	5.7%	39,584	2.6%	Lower			
		−2.4–0%	693	5.7%	40,711	2.7%	Lower			
		0.01–5%	1528	12.5%	81,108	5.4%	Higher	15.9%	6.6%	
		>5%	405	3.3%	17,986	1.2%	Higher			
