# Correction: Standen et al. Aboriginal Population and Climate Change in Australia: Implications for Health and Adaptation Planning. *Int. J. Environ. Res. Public Health* 2022, *19*, 7502

**DOI:** 10.3390/ijerph192416378

**Published:** 2022-12-07

**Authors:** Jeffrey C. Standen, Jessica Spencer, Grace W. Lee, Joe Van Buskirk, Veronica Matthews, Ivan Hanigan, Sinead Boylan, Edward Jegasothy, Matilde Breth-Petersen, Geoffrey G. Morgan

**Affiliations:** 1 Health Protection NSW, St Leonards, NSW 2065, Australia; 2 School of Public Health, Faculty of Medicine and Health, University of Sydney, Camperdown, NSW 2006, Australia; 3 University Centre for Rural Health, Faculty of Medicine and Health, University of Sydney, Lismore, NSW 2480, Australia

## Error in Figures 2c and 3a

In the original publication [1], there was an error in Figure 2c containing a map of climate exposures with bar charts indicating relative exposure by category across Aboriginal and non-Aboriginal populations. The exposures in Figure 2c were projected additional days exceeding 35 °C annually, 2020–2039. There was also an error in Figure 3a containing a map of annual days with Macarthur Forest Fire Danger Index exceeding 50 (i.e., “severe” fire danger), with bar charts indicating relative exposure by category across Aboriginal and non-Aboriginal populations for historical data between 1990 and 2009. During publication, formatting changes of the accepted manuscript occurred. 

The categories in the bar charts in Figure 2c and Figure 3a were incorrect. The corrected Figure 2 and Figure 3 appear below. There are no changes to the text in the manuscript.

**Figure 2:**
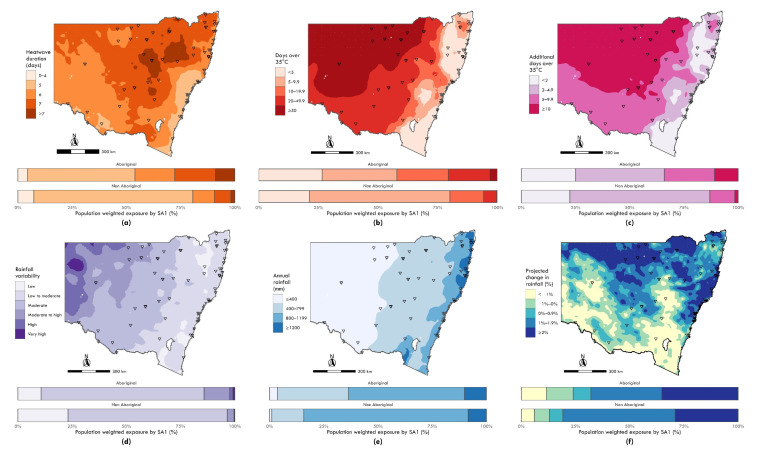
Maps of climate exposures with bar charts indicating relative exposure by category across Aboriginal and non-Aboriginal populations. Exposures include: (**a**) Historical annual average maximum heatwave duration (days), 1990–2019; (**b**) Historical annual days exceeding 35 °C, 1990–2019; (**c**) Projected additional days exceeding 35 °C annually, 2020–2039; (**d**) Historical annual rainfall variability, 1990–2019; (**e**) Historical annual rainfall in millimetres (mm), 1990–2019; (**f**) Projected relative change in annual rainfall, 2020–2039. Triangle markers denote identified discrete Aboriginal communities. See Appendix A for a summary of descriptive statistics for selected climate exposure estimates on a continuous scale.

**Figure 3:**
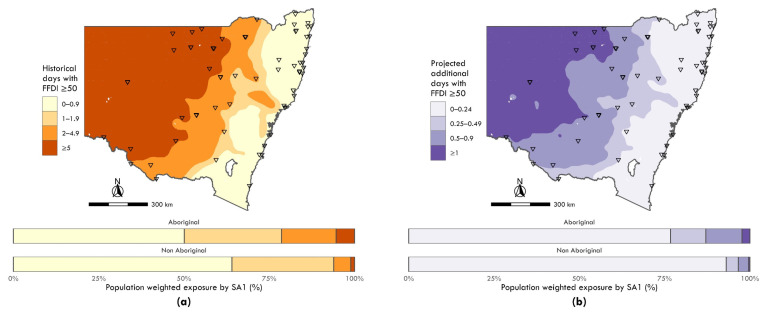
Maps of annual days with Macarthur Forest Fire Danger Index exceeding 50 (i.e., “severe” fire danger), with bar charts indicating relative exposure by category across Aboriginal and non-Aboriginal populations: (**a**) historical between 1990 and 2009; and (**b**) projected for 2020–2039. See Appendix A for a summary of descriptive statistics.

## Error in Table A2

In the original publication, there was a formatting error in Table A2 containing climate exposure estimates by Aboriginal versus non-Aboriginal usual resident populations stratified by the Index of Relative Socioeconomic Disadvantage (IRSD). The IRSD quintile 5 for the relative change in drought-affected months (1990–2006 vs. 2007–2020) was included in the online version but omitted from the pdf version of the published manuscript. 

The relevant section of the corrected Table A2 appears below.

**Table A2:** Climate exposure estimates by Aboriginal versus non-Aboriginal usual resident populations stratified by the Index of Relative Socioeconomic Disadvantage (IRSD). Small cell counts for either population have been aggregated.

Climate Exposure	IRSD Quintile	Exposure Category	Aboriginal URP		Non-Aboriginal URP		Binary Risk Category	High-Risk Exposure Population (%)		Odds Ratio
			n	%	n	%		Aboriginal	Non-Aboriginal	
Relative change in drought-affected months (1990–2006 vs. 2007–2020)	1	≤−10%	13,856	15.3%	344,184	23.3%	Lower			2.12 [2.09–2.15]
		−9.9–−5%	25,938	28.6%	609,131	41.2%	Lower			
		−4.9–−2.5%	9507	10.5%	135,713	9.2%	Lower			
		−2.4–0%	8878	9.8%	80,167	5.4%	Lower			
		0.01–5%	17,490	19.3%	178,228	12.1%	Higher	35.9%	20.9%	
		>5%	15,134	16.7%	130,805	8.8%	Higher			
	2	≤10%	8460	18.5%	418,629	30.7%	Lower			1.76 [1.72–1.79]
		−9.9–−5%	13,802	30.2%	452,136	33.1%	Lower			
		−4.9–−2.5%	4331	9.5%	109,654	8.0%	Lower			
		−2.4–0%	3622	7.9%	77,300	5.7%	Lower			
		0.01–5%	10,035	22.0%	202,484	14.8%	Higher	33.9%	22.5%	
		>5%	5427	11.9%	105,395	7.7%	Higher			
	3	≤−10%	6724	23.1%	459,306	38.2%	Lower			1.70 [1.66–1.75]
		−9.9–−5%	8323	28.6%	365,938	30.4%	Lower			
		−4.9–−2.5%	3383	11.6%	87,006	7.2%	Lower			
		−2.4–0%	2549	8.8%	68,078	5.7%	Lower			
		0.01–5%	5718	19.7%	153,570	12.8%	Higher	27.9%	18.5%	
		>5%	2401	8.3%	69,538	5.8%	Higher			
	4	≤−10%	6010	29.9%	526,829	45.0%	Lower			1.89 [1.83–1.96]
		−9.9–−5%	5613	27.9%	335,048	28.6%	Lower			
		−4.9–−2.5%	1503	7.5%	61,476	5.3%	Lower			
		−2.4–0%	1847	9.2%	66,667	5.7%	Lower			
		0.01–5%	3834	19.0%	134,397	11.5%	Higher	25.6%	15.4%	
		>5%	1324	6.6%	45,693	3.9%	Higher			
	5	≤−10%	4192	34.4%	645,501	43.2%	Lower			2.65 [2.53–2.79]
		−9.9–−5%	4677	38.4%	669,733	44.8%	Lower			
		−4.9–−2.5%	697	5.7%	39,584	2.6%	Lower			
		−2.4–0%	693	5.7%	40,711	2.7%	Lower			
		0.01–5%	1528	12.5%	81,108	5.4%	Higher	15.9%	6.6%	
		>5%	405	3.3%	17,986	1.2%	Higher			

The authors apologize for any inconvenience caused and state that the scientific conclusions are unaffected. This correction was approved by the Academic Editor. The original publication has also been updated.
